# Subsequent perinatal outcomes of pregnancy with two consecutive pregnancies with gestational diabetes mellitus: A population‐based cohort study

**DOI:** 10.1111/1753-0407.13263

**Published:** 2022-04-03

**Authors:** Yanni Guo, Xia Xu, Weijiao Xu, Tingting Liao, Jie Liang, Jianying Yan

**Affiliations:** ^1^ College of Clinical Medicine for Obstetrics & Gynecology and Pediatrics, Fujian Medical University Fuzhou China; ^2^ Fujian Maternity and Child Health Hospital Fuzhou China

**Keywords:** maternal and neonatal outcomes, recurrent GDM, 复发性妊娠期糖尿病, 母婴结局

## Abstract

**Background:**

Gestational diabetes mellitus (GDM) is glucose intolerance diagnosed during pregnancy. We aimed to explore the different outcomes of women with two consecutive pregnancies with GDM.

**Methods:**

This study included 861 women with recurrent GDM who had two consecutive singleton deliveries at Fujian Maternity and Child Health Hospital between May 2012 and September 2020. Data on pregnancy complications and neonatal and delivery outcomes were collected and analyzed.

**Results:**

Among those women with recurrent GDM, there was no difference in pregnancy complications in index pregnancy vs subsequent pregnancy. Our data revealed there was a significantly higher incidence of thyroid disease in the subsequent pregnancies than in the index pregnancy. (6% vs 10%, *p* = .003)In subsequent pregnancies, the birth weight was greater than that of the index pregnancy (3296.63 ± 16.85 vs 3348.99 ± 16.05, *p* = .025); and the incidence of large for gestational age (LGA) was higher than that of the index pregnancy (16.3% vs 20.6%, *p* = .021). More cesarean sections occurred in the subsequent pregnancy. (32.9% vs 6.6%, *p* = .039). Postpartum hemorrhage, premature birth, and placental abruption were not significantly different between the two pregnancies.

**Conclusions:**

The results suggest the effect of GDM on thyroid dysfunction may be persistent. Recurrent gestational diabetes results in a higher rate of cesarean delivery, incidence of LGA, and neonatal admission to the neonatal intensive care unit (NICU) in subsequent pregnancies. We need to pay attention to the postpartum thyroid function of pregnant women with GDM. Further studies are still needed on recurrent GDM to reduce this occurrence of admission to NICU.

## Graphical abstract

Highlights

To summarize, we found that the incidence of complications in subsequent pregnancies in women with recurrent GDM did not differ from that in their index pregnancies. There is a high incidence of thyroid disease in women with recurrent GDM in this study. And whether thyroid disorders increase in women with a history of GDM needs to be confirmed by further studies. The main reasons for cesarean delivery in women with uncomplicated GDM were fetal distress, macrosomia, and failed induction of labor. Therefore, prenatal guidance and the pre‐delivery evaluation of women with their first incidence of GDM are especially important and need careful clinical management. There is a high rate of neonatal admission to NICU in cases of recurrent GDM, mainly due to neonatal respiratory disease. This study had some limitations. Firstly, our study is a single‐center observational cohort study with a moderate sample size and no control group. Secondly, these changes can not be ruled out due to the possibility of age. Further research is required to investigate how we can reduce this occurrence.

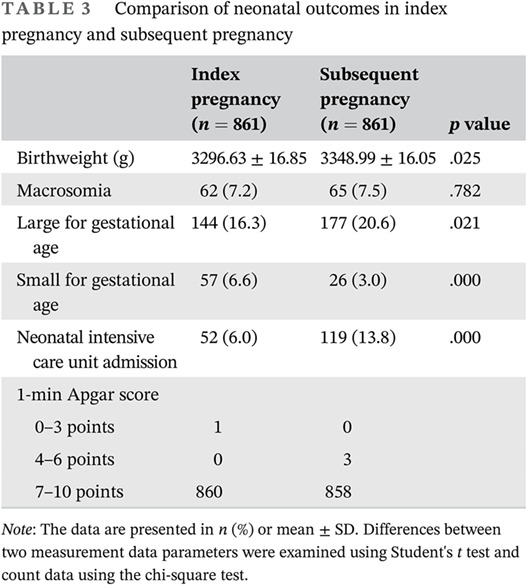

## 摘要

**背景:**

妊娠期糖尿病(GDM)是妊娠期确诊的糖耐量异常。我们的目的是探索连续两次妊娠的GDM妇女的不同结局。

**方法:**

研究对象为自2012年5月至2020年9月在福建省妇幼保健院连续两次单胎分娩的复发性GDM患者861例。收集并分析妊娠并发症, 新生儿和分娩结局。

**结果:**

在复发性GDM患者中, 首次妊娠与再次妊娠在妊娠并发症方面无明显差异。我们的数据显示, 再次妊娠时甲状腺疾病的发病率明显高于首次妊娠(6% vs 10%,p=0.003)。再次妊娠组新生儿出生体重高于首次组(3296.63±16.85g vs 3348.99±16.05g, p=0.025), 大于胎龄儿(LGA)高于首次组(16.3% vs 20.6%, p=0.021)。再次妊娠时发生更多的剖腹产(32.9% vs 6.6%, P=0.039)。两组在产后出血, 早产, 胎盘早剥的差异无统计学意义。

**结论:**

本研究结果提示GDM对甲状腺功能障碍的影响可能是持久的。复发的妊娠期糖尿病具有较高的剖宫产率, LGA发生率, 以及更多的进入新生儿重症监护病房(NICU)的新生儿。GDM孕妇的产后甲状腺功能有待进一步研究, 以降低NICU的发生率。

## 1 BACKGROUND

Gestational diabetes mellitus (GDM) is a disease that is specific to pregnancy and is defined by abnormal glucose tolerance that occurs to varying degrees during pregnancy. We adopted the diagnostic criteria proposed by the International Association of Diabetes and Pregnancy Study Group. Pregnant females with gestational diabetes are considered to have a high‐risk pregnancy, with an increased incidence of adverse maternal and child outcomes, such as preeclampsia, amniotic fluid abnormalities, neonatal hypoglycemia, macrosomia, and obstructed shoulder birth; this also increases the risk of GDM in subsequent pregnancies.^1^, ^2^ Research has shown that the probability of recurrence of gestational diabetes varies from 30% to 84%.^3^ Recurrent GDM is defined as a history of GDM and a subsequent pregnancy with abnormal glucose tolerance along with indicators that satisfy the diagnostic criteria for GDM. The duration of breastfeeding, pregnancy interval, weight change, and ethnicity have different effects on the recurrence rate of gestational diabetes.^4^, ^5^, ^6^ Thyroid function and glucose metabolism are in an interactive relationship. Studies have shown that thyroid dysfunction in early pregnancy is associated with an increased risk of developing GDM. Overt thyroid dysfunction is known to occur in almost 1% of all pregnant women, and there are also studies that suggest that maternal hyperglycemia is a risk factor for the development of thyroid autoimmunity.^8^ The full opening of the two‐child policy in China in 2015 has increased the number of people with recurrent GDM as the number of people choosing to have another pregnancy increases. There are few studies related to recurrent GDM. Consequently, the purpose of this cohort study was to examine two deliveries before and after in pregnant women with recurrent GDM and to investigate the differences between recurrent GDM and initial GDM.

## 2 METHODS

This cohort study involved the analysis of women who had two consecutive singleton deliveries at Fujian Maternity and Child Health Hospital between May 2012 and September 2020. Patients were included if there was a diagnosis of GDM during both pregnancies. All patients followed diet and lifestyle recommendations made upon after the initial diagnosis of GDM. Patients were excluded if they (a) had their first delivery earlier than May 2012 or the first delivery occurred at another hospital, (b) had diabetes after their first pregnancy or in the first trimester of their second pregnancy, (c) had experienced more than two deliveries during the observation period, (d) y had experienced stillbirth or late miscarriage in one of the pregnancies, and (e) had multiple pregnancies. The control standard levels for blood glucose during pregnancy were a finger‐prick fasting blood glucose >3.3–5.3 mmol/L, 1‐hour postprandial blood glucose <7.8 mmol/L, and 2‐hour postprandial blood glucose <6.7 mmol/L.^9^ Insulin was used when the glycemic goals were not achieved after 1 to 2 weeks.

**FIGURE 1:**
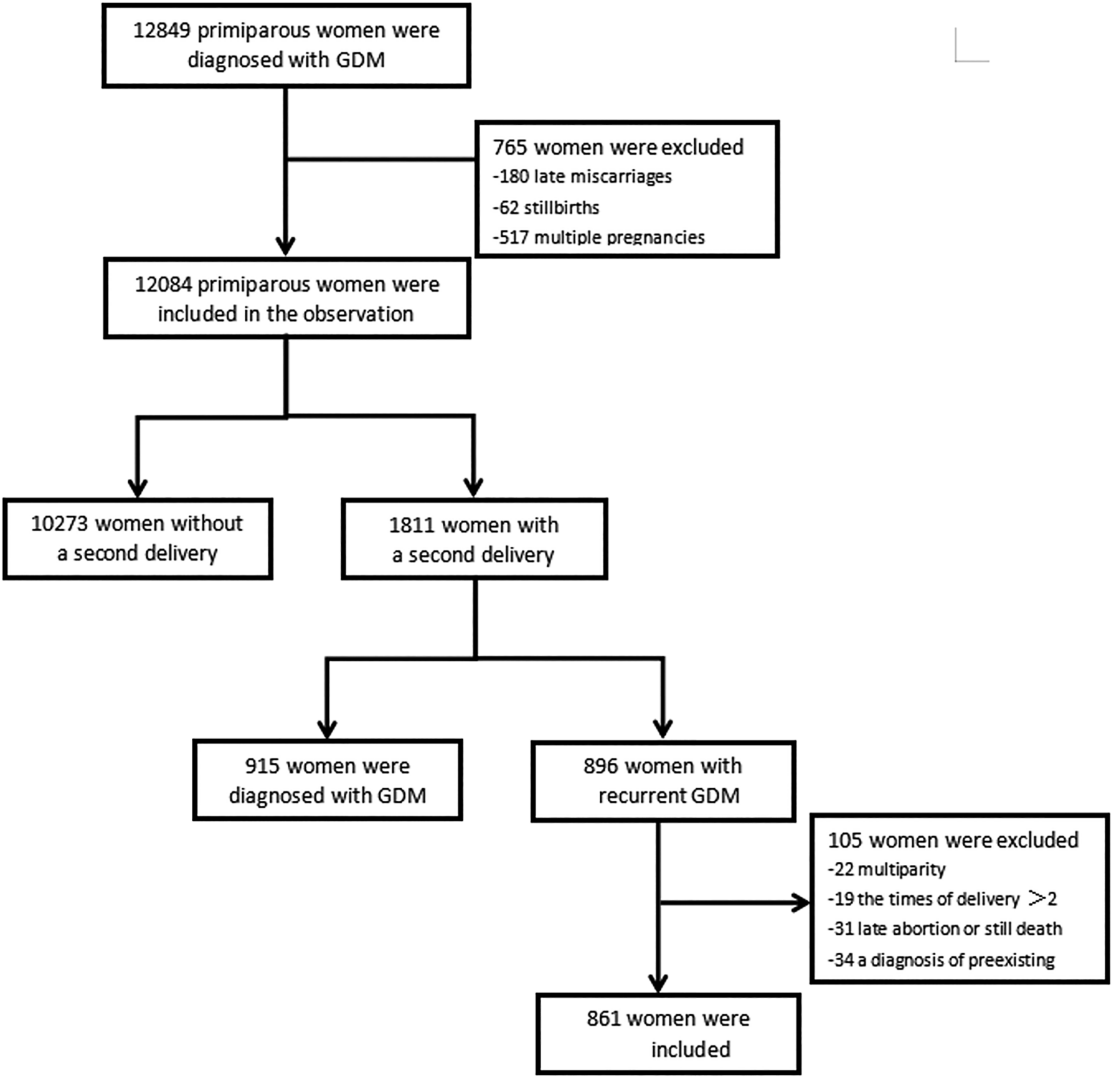
Flow chart of the study population. GDM, gestational diabetes mellitus

Figure 1 shows that a total of 12 849 primiparous women were diagnosed with GDM between May 2012 and September 2020 at Fujian Maternal and Child Health Hospital. Of these, we excluded 180 late miscarriages, 62 stillbirths, and 517 multiple pregnancies. A total of 1811 women had a second pregnancy and delivered in our hospital during the observation period, and 34 women were excluded during the observation period because of prepregnancy diabetes. Finally, 896 women had recurrent gestational diabetes. Of these, we excluded 7 cases involving late miscarriage, 9 cases of multiple pregnancy, and 20 cases because of a third delivery. In our study, early miscarriage was defined as fetal delivery at <12 weeks of gestational age. Late miscarriage was defined as fetal delivery at <24 weeks of gestational age. In China, once a diagnosis of COVID‐19 has been confirmed, the patient will be seen in a designated hospital; therefore, this study featured no pregnant women with COVID‐19.

A total of 861 women were included in this study; from each patient, we collated a range of data, including age, mode of conception, time of last menstrual period, amniotic fluid volume, time between deliveries, gestational weeks of delivery, pregnancy complications, neonatal weight, whether they were admitted to neonatal intensive care unit (NICU) and the reasons for this, mode of delivery, number of days in hospital, and cost. We also determined the difference between the time of delivery for the index pregnancy and the time of the last menstrual period of the subsequent pregnancy. Preexisting hypertension, pregnancy‐induced hypertension, and preeclampsia were defined as hypertensive disorders. The thyroid diseases described in this article include hypothyroidism and hyperthyroidism. We diagnosed thyroid diseases and thyroiditis based on a physician diagnosis documented in medical records. According to the medical records, before June 2017 we used the 2011 guidelines put forward by the American Thyroid Association (ATA). After June 2017, we used the 2017 ATA guidelines to diagnose thyroid diseases and thyroiditis.^10^, ^11^ Birth weight was measured immediately after delivery. A birthweight >4000 g was defined as macrosomia. Large for gestational age (LGA) fetuses were diagnosed when the weight exceeded the 90th percentile for gestational age (gestational age‐standardized birth weights). Small for gestational age (SGA) fetuses were diagnosed when the weight fell below the 10th percentile for gestational age.^12^ Preterm birth was defined as less than 37 weeks of gestation. For this study, respiratory diseases included respiratory distress syndrome, transient tachypnea, meconium aspiration, pneumothorax, pneumonia, or apnea.

We used SPSS version 23.0 (SPSS Inc., Chicago, IL) for data analysis. The *t* test was used to analyze quantitative data and chi‐square test or Fisher's exact test was used for qualitative categorical material. All statistical tests were performed with a two‐sided *p* value. Differences were considered statistically significant if the *p* value was <.05. The procedures followed were in accordance with the ethical standards of the responsible committee on human experimentation (institutional or regional) and with the Helsinki Declaration of 1975, as revised in 2000. An exemption was granted from the requirement for informed consent because the study had a retrospective design.

## 3 RESULTS

Table 1 shows the clinical profile of the two pregnancies for each case. The age of the previous pregnancy was 28.52 ± 3.33 years old and the age of the subsequent pregnancy was 31.82 ± 3.57 years old; 198 cases were of advanced maternal age in their subsequent pregnancy; this was much higher than the number of cases in the first pregnancy (33). The gravidity of index pregnancies was 1.48 ± 0.027 whereas that of the subsequent pregnancies was (2.64 ± 0.029); the interval between pregnancies was 31.59 ± 17.58 months. A significantly higher number of women used assisted reproduction for their first pregnancy than their second pregnancy (3.1% vs 1.2%, *p* = .005).

**TABLE 1:** Clinical characteristics

	Index pregnancy (*n* = 861)	Subsequent pregnancy (*n* = 861)	*p* value
Age, years	28.52 ± 3.33	31.82 ± 3.57	
Age ≥ 35 years old	33 (3.8)	198 (23)	.000
Assisted reproductive techniques	27 (3.1)	10 (1.2)	.005
Gravidity	1.48 ± 0.027	2.64 ± 0.029	
Interval between pregnancies, months	31.69 ± 17.58		

*Note*: The data are presented in *n* (%) or mean ± SD. Differences between two measurement data parameters were examined using Student's *t* test and count data using the chi‐square test.

Table 2 shows that there were no significant differences between the two pregnancies in terms of the incidence of hypertensive disorders of pregnancy, intrahepatic cholestasis of pregnancy, and abnormal amniotic fluid volume, although the incidence of intrauterine distress was significantly higher in the first pregnancy than in the subsequent pregnancy (*p* = .000). Our data revealed that there was a significantly higher incidence of thyroid disease in the subsequent pregnancies than in the index pregnancy (6% vs 10%; *p* = .003). The thyroid diseases mentioned in this article include hyperthyroidism and hypothyroidism. There were four women with hypothyroidism and two with hyperthyroidism before the index pregnancy. There were two women with hypothyroidism and six with hyperthyroidism between two deliveries. Moreover, the incidence of thyroiditis is higher in subsequent pregnancies than in the index pregnancies. (1.4% vs 3.5%; *p* = .005).

**TABLE 2:** Pregnancy complications for women with recurrent gestation diabetes mellitus

	Index pregnancy (*n* = 861)	Subsequent pregnancy (*n* = 861)	*p* value
Gestational hypertensive disorders	60 (7.0)	45 (5.2)	.131
Gestational hypertension	26 (3)	15 (1.7)	.082
Preeclampsia	30 (3.5)	25 (2.9)	.493
Eclampsia	0	0	‐
Chronic hypertension complicating pregnancy	1 (0.1)	5 (0.6)	.218
Chronic hypertension with superimposed preeclampsia	3 (0.3)	5 (0.6)	.726
Intrahepatic cholestasis of pregnancy	0	5 (0.6)	.073
Abnormal amniotic fluid	49 (5.8)	33 (3.8)	.117
Polyhydramnios	22 (2.6)	13 (1.5)	.124
Oligoamnios	28 (3.3)	20 (2.3)	.242
Thyroid dysfunction	41 (4.8)	61 (7.1)	.041
Hypothyroidism or subclinical hypothyroidism	23 (2.7)	36 (4.2)	.085
Hyperthyroidism or subclinical hyperthyroidism	18 (2.1)	25 (2.9)	.280
Thyroiditis	12 (1.4)	30 (3.5)	.005
Fetal distress	71 (8.2)	18 (2.1)	.000

*Note*: The data are presented in *n* (%) or mean ± SD. Differences between two measurement data parameters were examined using Student's *t* test and count data using the chi‐square test.

The neonatal outcomes for both pregnancies are described in Table 3. The weight of the newborn from the subsequent pregnancy was significantly higher than the weight of the newborn from the index pregnancy (*p* = .025), although there was no significant difference in the incidence of macrosomia when compared between the two pregnancies (*p* = .782). However, the incidence of LGA was significantly higher in subsequent pregnancies than in index pregnancies, and the results were statistically significant. (16.3% vs 20.6%; *p* = .021) and the incidence of SGA was lower. (6.6% vs 3.0%; *p* = .000). Among the neonatal outcomes, the rates of NICU admission in subsequent pregnancies were significantly higher than those in the previous pregnancy (*p* = .000).

**TABLE 3:** Comparison of neonatal outcomes in index pregnancy and subsequent pregnancy

	Index pregnancy (*n* = 861)	Subsequent pregnancy (*n* = 861)	*p* value
Birthweight (g)	3296.63 ± 16.85	3348.99 ± 16.05	.025
Macrosomia	62 (7.2)	65 (7.5)	.782
Large for gestational age	144 (16.3)	177 (20.6)	.021
Small for gestational age	57 (6.6)	26 (3.0)	.000
Neonatal intensive care unit admission	52 (6.0)	119 (13.8)	.000
1‐min Apgar score			
0–3 points	1	0	
4–6 points	0	3	
7–10 points	860	858	

*Note*: The data are presented in *n* (%) or mean ± SD. Differences between two measurement data parameters were examined using Student's *t* test and count data using the chi‐square test.

Of the delivery outcomes shown in Table 4, the probability of meconium‐stained amniotic fluid in the index pregnancy was significantly higher than in the subsequent pregnancy (*p* = .000), although the incidence of bloody amniotic fluid was significantly higher in the subsequent pregnancy (*p* = .000). With regard to the choice of delivery method, the incidence of cesarean delivery was significantly higher in subsequent pregnancies (*p* = .039). There was no significant difference between the two pregnancies with regard to the incidence of postpartum hemorrhage, premature birth, or placental abruption. However, there was a significantly higher incidence of premature rupture of membranes in the first pregnancy (*p* = .000). In terms of the number of gestational weeks at delivery, subsequent pregnancies delivered significantly earlier than the index pregnancies (*p* = .000). Interestingly, we found that subsequent pregnancies had higher hospitalization costs than previous pregnancies (*p* = .000), but shorter hospitalization days (*p* = .000).

**TABLE 4:** Comparison of delivery outcomes in index pregnancy and subsequent pregnancy

	Index pregnancy (*n* = 861)	Subsequent pregnancy (*n* = 861)	*p* value
Meconium‐stained amniotic fluid	159 (18.5)	109 (12.7)	.000
Bloody amniotic fluid	0 (0)	22 (2.6)	.000
Cesarean section	283 (32.9)	324 (37.6)	.039
Postpartum hemorrhage	25 (2.9)	26 (3.0)	.887
Precipitate delivery	27 (3.1)	177 (20.5)	.000
Premature birth	55 (6.39)	73 (8.48)	.098
Placental abruption	8 (0.9)	17 (2.0)	.070
Premature rupture of membranes	251 (29.2)	135 (15.7)	.000
Week of delivery	39.26 ± 0.05	38.79 ± 0.07	.000
Hospitalization expenses (yuan)	6730.86 ± 109.74	8055.78 ± 185.30	.000
Length of hospitalization (days)	5.56 ± 2.58	4.72 ± 5.98	.000

*Note*: The data are presented in *n* (%) or mean ± SD. Differences between two measurement data parameters were examined using Student's *t* test and count data using the chi‐square test.

## 4 DISCUSSION

Previous studies have shown that the incidence of type 2 diabetes mellitus (T2DM) after gestational diabetes varies widely, ranging from 1.3% to 70%.^7^ When the mean follow‐up time was extended to 4.40 years, the incidence rate of diabetes was 24.7/1000 person‐years in GDM women and the mean age was 30.1 years.^13^ The incidence of T2DM in subjects with a history of GDM is closely related to the duration of follow‐up, race, body mass index, and age. The mean interval between the pregnancies in this study was ~2.64 years, and the mean age at the index pregnancy is 28.52 years, which are factors in the incidence of T2DM.

According to our study, there was a significant difference in the incidence of pregnancy complications among women with recurrent gestational diabetes. In a previous study, the incidence of preterm delivery subsequent pregnancy was higher than that of index pregnancy^14^; however, in the present study, we found no difference. Previous studies have reported a higher incidence of thyroid disorders in pregnant women with GDM than in healthy individuals.^15^ One previous study reported that hypothyroidism was associated with the occurrence of GDM.^16^ The relationship between thyroid disorders and GDM is controversial. Thyroid dysfunction and positive thyroid antibodies are associated with the risk of gestational diabetes.^17^ Studies have shown that lower free thyroid hormone levels are associated with higher blood sugar and insulin resistance. Furthermore, the improvements in blood glucose and insulin resistance were accompanied by the restoration of lownormal thyroid function.^18^ The study showed that the levels of sirtuin 1 and sirtuin 3 were lower in peripheral blood samples from patients with type 1 diabetes, T2D, or hypothyroidism than in healthy individuals. This study observed alterations in the expression levels of sirtuins and superoxide dismutase in diabetes and hypothyroidism, which may be related to oxidative stress.^19^ Oxidative stress generally promotes the inflammatory response. There is a study indicating that elevated levels of thyroid antibodies increase the release of inflammatory factors, which can lead to insulin resistance and diabetes.^20^ During pregnancy and the postpartum period, autoimmunity is the most common cause of the occurrence of thyroid dysfunction.^21^ In the present study, the incidences of thyroid disorders and thyroiditis were higher in subsequent pregnancies (*p* = .003 and *p* = .005). A previous study reported that maternal GDM exerts effect on the human placental thyroid hormone receptor (THR) subtypes THRα1, THRα2, THRβ1, and THRβ2.^22^ We speculate that this effect may be persistent if a woman with GDM became pregnant again. The association between thyroid autoimmunity and spontaneous type 1 diabetes has been well established by numerous studies.^23^ The most common form of autoimmune disease in diabetes patients is autoimmune thyroiditis; this can lead to thyropathy.^24^ In addition, a previous study has described the coexistence of immune checkpoint inhibitor‐induced thyroiditis and autoimmune diabetes mellitus.^25^ The occurrence of recurrent gestational diabetes and thyroid dysfunction may interact with each other.

A previous study reported that the incidence of cesarean delivery was higher in subsequent pregnancies (*p* = .009).^14^ In total, 283 women who had a cesarean delivery with their first pregnancy still opted for a second cesarean delivery in their subsequent delivery; their mean gestational week of delivery was 38.44 weeks. This is one of the reasons why subsequent pregnancies are delivered at smaller number of gestational weeks than the index pregnancy. 18 of these women opted for a trial of vaginal delivery; in the present study, we found that the use of forceps was significantly more common for the first delivery than the second delivery (1.5% vs 0.3%, *p* = .000). A previous study reported a higher incidence of emergency cesarean delivery in pregnant women with uncomplicated GDM than in normal pregnant women.^26^ The rate of cesarean delivery in our study was 32.9% in index cases of GDM; forceps were also used more commonly. The main reasons for cesarean delivery were fetal distress, macrosomia, failed induction of labor, and an abnormal fetal position. The number of index pregnancies that underwent emergency cesarean section because of fetal distress was 58. Therefore, it is important that we fully consider t weight management and guidance during pregnancy for women with first‐time GDM and pay close attention to the progress of labor and the occurrence of precipitate delivery in women with recurrent GDM.

In the present study, the mean number of gestational weeks for elective repeat cesarean delivery was 38.44 weeks. This may explain the shorter number of gestational weeks for subsequent pregnancies when compared to previous pregnancies. We showed that the mean number of days of hospital stay was shorter in subsequent pregnancies; this may be owing to the higher rate of emergency deliveries and cesarean deliveries in subsequent pregnancies. Currently we adopt the single disease payment system in our institution. A single disease payment is a projection of the cost of medical care for the entire process of treating a single disease, with a corresponding payment measure. The cost‐control effect of the single‐patient payment is remarkable, as shown by a significant reduction in hospital days and drug costs.^27^ Therefore, the length of stay is not the main determinant of hospital costs. In addition, over the last two years, we have started to use rapid recovery for cesarean delivery and rapid recovery for vaginal delivery, which can reduce the number of hospital days by 1 to 2 days compared to traditional postpartum recovery.

The color of the amniotic fluid (clear, meconium stained, bloody) during labor is determined by the obstetricians'/midwives’ subjective impression. A longer duration of labor was identified as a key factor associated with an increased risk for meconium‐stained amniotic fluid. Evidence has shown that the incidence of meconium‐stained fluid increases as the gestational age increases.^28^, ^29^ The incidence of meconium‐stained amniotic fluid is higher in index pregnancies because of the increased number of weeks of gestation and the long duration of the first delivery. We do not know why this happens if labor was not associated with placental abruption; the clear reason behind this is still unknown. Bloody amniotic fluid during labor was associated with higher rates of labor induction, assisted vaginal deliveries, and cesarean deliveries.^30^ The birth weight of subsequent pregnancies is greater than that of the index pregnancy. A previous study has also shown that multiparity is a risk factor for the development of LGA.^31^

We also found that the probability of neonatal admission to the NICU in subsequent pregnancies of women with recurrent GDM was much higher than in previous pregnancies. The main causes of NICU admission in subsequent pregnancies were neonatal respiratory diseases (71/119, 59.67%) and neonatal hypoglycemia (17/119, 14.29%); two children were smaller than their gestational age (4/119, 3.36%). The main causes of NICU admission in index pregnancies were neonatal respiratory diseases (33/52, 63.46%). Neonatal respiratory disorders are considered to represent a major cause of neonatal morbidity and mortality and require early detection, diagnosis, and treatment. The relationship between maternal diabetes and neonatal respiratory distress syndrome has been recognized for some time; however, research findings associated with this relationship are inconsistent. For example, Baseer and Kawakita et al reported that diabetes is one of the most important risk factors associated with neonatal respiratory disorders,^32^, ^33^ whereas Bricelj et al held the opposite opinion.^34^

Previous studies have shown that the potential damage to beta cells from GDM persists after the first pregnancy.^35^ Women with a history of GDM are therefore more likely to develop insulin resistance in a nonpregnant state, and insulin sensitivity is further reduced.^36^, ^37^ A study by Miakotina et al found that insulin reduced the expression of surface‐active protein A and surface‐active protein B, thus affecting fetal lung development.^38^ The potential damage caused by GDM persists after the initial pregnancy; this damage may be further exacerbated in subsequent pregnancies, thus leading to an increased incidence of neonatal respiratory disease. A retrospective study by Paul et al showed that the incidence of respiratory disease was reduced if glucocorticoids were used prenatally in women who delivered by cesarean section after 37 weeks of gestation.^39^ Further evidence is now needed to ascertain whether women with recurrent diabetes need to use glucocorticoids before delivery to further promote fetal lung maturation.

To summarize, there is a high incidence of thyroid disease in women with recurrent GDM in this study. Whether thyroid disorders increase in women with a history of GDM needs to be confirmed by further studies. Based on the findings of this study, we recommend that pregnant women with a history of GDM be monitored for thyroid function and antibodies in the postpartum period and in the early stages of subsequent pregnancies. The main reasons for cesarean delivery in women with uncomplicated GDM were fetal distress, macrosomia, and failed induction of labor. Therefore, prenatal guidance and the predelivery evaluation of women with their first incidence of GDM are especially important and need careful clinical management. There is a high rate of neonatal admission to NICU in cases of recurrent GDM, mainly owing to neonatal respiratory disease.

This study had some limitations that need to be considered. First, our study is a single‐center observational cohort study with a moderate sample size and no control group. Second, these changes cannot be ruled out as being due to age factors. Third, one of the limitations of this study was that it is a single‐center retrospective study; the sample size was small, and the follow‐up period was short. Consequently, the power of this study may not have been sufficient to identify differences macrosomia. Certainly, it would be better to carry out detailed of analysis of populations requiring insulin therapy for recurrent GDM. We have already initiated such research. Further research is now required to investigate how we can reduce this occurrence.

## CONFLICT OF INTEREST

The authors declare that they have no conflict of interest and funding.

## AUTHOR CONTRIBUTIONS

All authors contributed to manuscript editing and read and approved the final manuscript. Yanni Guo and Xia Xu contributed to the study design. The analysis was made by Yanni Guo in discussion and with the assistance of Xia Xu, Tingting Liao, and Jie Liang. Yanni Guo, Xia Xu, Weijiao Xu and Tingting Liao drafted the manuscript. Yanni Guo and Xia Xu equally contributed to the work.

## CONSENT FOR PUBLICATION

Not applicable.

## ETHICS APPROVAL AND CONSENT TO PARTICIPATE

Ethical approval was obtained from the Fujian Maternity and Child Health Hospital Ethics Committee (2020‐2049). All human studies have been reviewed by the appropriate ethics committee and have therefore been performed in accordance with the ethical standards laid down in an appropriate version of the Declaration of Helsinki (as revised in Brazil 2013). Details that might disclose the identity of the subjects under the study should be omitted. Photographs need to be cropped sufficiently to prevent human subjects being recognized (or an eye bar should be used). The submission of a case report should be accompanied by the written consent of the subject (or parent/guardian) before publication. The photographs are to be used or in cases where the unique nature of the incident reported makes it possible for the patient to be identified.

## Data Availability

The data sets used and analyzed during the current study are available from the corresponding author on reasonable request.
